# Fatal asphyxia due to large laryngeal granuloma

**DOI:** 10.4322/acr.2024.496

**Published:** 2024-06-14

**Authors:** Ajay Kumar, Ravi Rautji, Asit Ranjan Mridha, Chittaranjan Behera

**Affiliations:** 1 All India Institute of Medical Sciences, Department of Forensic Medicine & Toxicology, New Delhi, Delhi, India; 2 Bharati Vidyapeeth Medical College, Department of Forensic Medicine & Toxicology, Pune, India; 3 All India Institute of Medical Sciences, Department of Pathology, New Delhi, Delhi, India

**Keywords:** Granulation Tissue, Fibrin, Granuloma laryngeal, Polyp, Asphyxia

## Abstract

Laryngeal granuloma, vocal process granuloma, or post-intubation granuloma are benign, inflammatory lesions of the arytenoid cartilage vocal process. The etiology of laryngeal granulomas is multifactorial, such as chronic irritation due to endotracheal intubation, vocal cord injury or trauma, and gastroesophageal reflux disease. They can arise postoperatively after mucosal injury due to orotracheal intubation. Clinical manifestations include voice change and dyspnea, which may start one to four months after extubation and may rarely lead to asphyxia. We presented a case of death due to glottic granuloma occurring after a surgical procedure to remove a laryngeal polyp attributed to previous laryngeal injuries by multiple intubations.

## INTRODUCTION

Laryngeal granuloma, first described by Chevalier Jackson in 1928 as a contact laryngeal ulcer, is commonly termed a laryngeal contact ulcer, contact granuloma, vocal process granuloma, vocal fold granuloma or post-intubation granuloma.^1-4^ Vocal process granulomas are benign, inflammatory lesions located over the vocal process of the arytenoid cartilage.^2^ They may be uni or bilateral ulcerative, pedunculated, sessile, erythematous, or white lesions. They often arise postoperatively following an injury of the arytenoid cartilage mucosa overlying.^1^ Clinical manifestations include voice change and dyspnea, which may not manifest until one to four months after extubation,^1^ and may lead to potentially fatal complications such as asphyxia. Vocal granulomas affect both genders^5^ and can be caused by acid laryngitis secondary to laryngopharyngeal reflux disease, vocal overuse, and traumatic or prolonged intubation.

The pathogenesis of laryngeal granuloma is not entirely clear. However, it is hypothesized to occur due to inflammation or mucosal ulceration at the insult site.^6^ The histopathology of laryngeal granuloma reveals squamous hyperplasia with a proliferation of capillaries, fibroblasts, collagen fibers, and leukocytes.^4,6^ Over some time, the granulation tissue may become excessive and exuberant, causing significant airway obstruction and compromising the respiratory function.

## CASE REPORT

A 34-year-old male suddenly developed difficulty to breathe during the night. He was planning to go to the hospital when he collapsed outside his house and was immediately taken to the nearby hospital. He reached the hospital in an unconscious and unresponsive state without palpable pulses and respiratory movements. ECG showed an isoelectric straight line, and he was declared as brought dead. Available treatment records and detailed history from the deceased relatives revealed that he had been having difficulty breathing for the last 5 to 6 years. He had first developed hoarseness 8 years back and was diagnosed as a case of vocal cord polyp for which micro-laryngoscopic (MLS) excision was done. MLS excision was done again 8 months later due to symptoms recurrence. The histopathology of the excised mass showed focal dysplasia with actinomycosis. Five months later, he again developed symptoms, and because of extensive supra-glottic, glottic, and sub-glottic mass, elective tracheostomy with MLS debulking was done using a micro-debrider. Histopathology of the mass revealed an inflammatory polyp. He again developed similar hoarseness a year later, and indirect laryngoscopy revealed recurrent vocal cord polyp/papilloma anteriorly in glottic and subglottic regions, which was managed by MLS excision.

He underwent surgical resection of vocal cord polyp multiple times over 8 years, the last one being a month before his demise. Three days before his death, he had gone to the hospital complaining of breathing difficulty, which was exacerbated by exertion. On examination, a polyp was noted on the anterior commissure, obliterating the anterior half of the glottic region. There was no history of hypertension, diabetes mellitus, bronchial asthma, or tuberculosis.

## AUTOPSY FINDINGS

**Figure 1:**
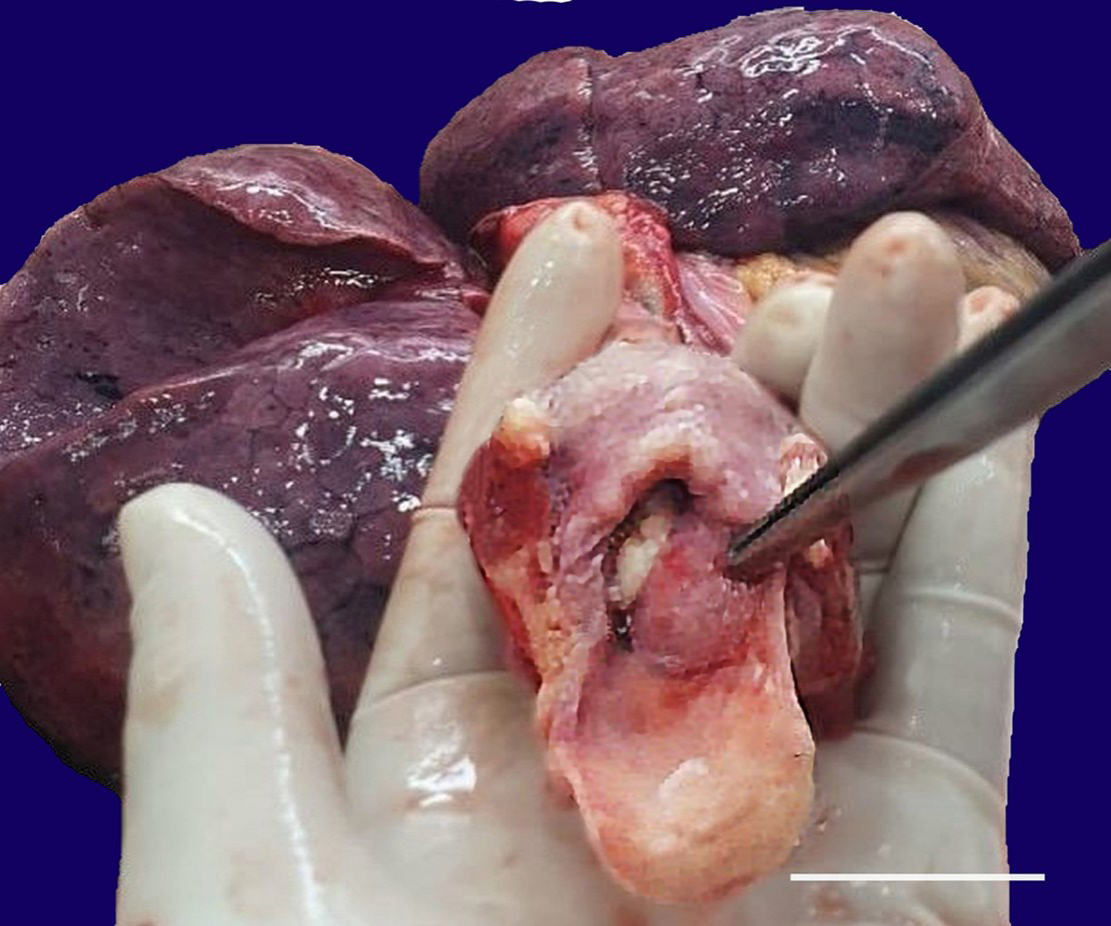
Gross view of a nodular mass measuring 3 cm x 2 cm occluding the laryngeal lumen (scale bar = 4 cm).

The deceased was a 34-year-old male of average build and height. Pre-autopsy Postmortem Computed Tomography showed a hyperdense mass in the larynx, obstructing almost three-fourths of the laryngeal lumen. On examination of the eyes, corneas were hazy, and conjunctivae showed congestion. Bluish discoloration of nails was noted. Examination of the oral cavity, oropharynx, and nasal cavity was unremarkable. The brain was congested, and diffuse petechial hemorrhages were present in the white matter. On the neck dissection, a nodular mass with a smooth surface, measuring 3 x 2 cm in its longest axis, was found attached to the anterior one-third of the left vocal cord extending up to the left pyriform fossa and sub-glottic region, completely occluding the laryngeal lumen (Figure 1).

The tracheobronchial tree contained blood-stained frothy fluid. Lungs were congested and edematous, weighing 620 g (RR:280-500 g) and 450 g (RR:240-340g) on the right and left side, respectively. The heart weighed 340 g (RR:233-383g). All the coronaries were patent, and heart walls, valves, and chambers showed no abnormality. The stomach contained about 150 ml of yellowish semi-digested food; gastric mucosa was normal. The liver, spleen, and kidneys were congested.

**Figure 2:**
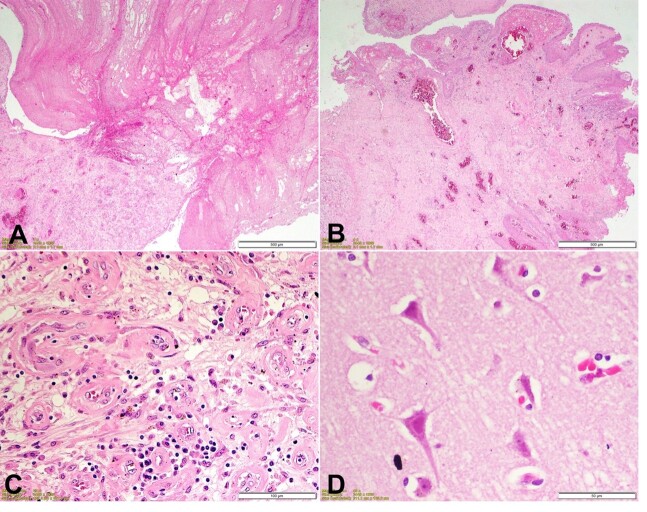
Photomicrographs of: **A** – section from polypoid mass shows mushroom-shaped fibrin clot (H&E, 40x); **B**  **–** An adjacent area near the base shows papillary proliferation with squamous metaplasia over the granulation tissue (H&E 40x); **C** **–** The base of the mass shows inflammatory granulation tissue with edema, proliferating capillaries, fibroblastic cells proliferation, lymphocytes, and plasma cells infiltrate (H&E 200x); **D** **–** Section from the brain shows red neurons with shrunken cytoplasm, angulated nuclei and uniform eosinophilia of cytoplasm and nuclei (H&E 400x).

Microscopic examination was done by a consultant pathologist in the Department of Pathology, AIIMS, New Delhi, for all the organs preserved during the autopsy. Section from polypoid mass showed a mushroom-shaped fibrin clot (Figure 2A) with an adjacent area showing papillary proliferation of mucosa with squamous metaplasia (Figure 2B). The base of the mass showed inflammatory granulation tissue with edema, proliferating capillaries, fibroblastic cell proliferation, lymphocytes, and plasma cell infiltrate (Figure 2C). Sections from the brain showed red neurons with shrunken cytoplasm, angulated nuclei, and uniform eosinophilia of cytoplasm and nuclei (Figure 2D).

The cause of death was asphyxia as a result of laryngeal obstruction due to complications of vocal polyp.

## DISCUSSION

In a PubMed search, we found one case after laryngeal neoplasm operation,^7^ two cases after vocal abuse/gastro-oesophageal reflux,^8^ one systemic review of post-intubation laryngeal granulomas of 85 patients.^9^ Our case is the first reported case of death due to glottic granuloma occurring after several surgical procedures for laryngeal polyp resection.

Vocal process granulomas are benign chronic inflammatory lesions that often develop near the posterior vocal cords, which are believed to arise after mechanical trauma or inflammation of the posterior glottis. They may mimic a malignant process by their enormous size, but the granulation tissue shows no tendency to malignant transformation.^10^

The etiology of laryngeal granuloma is multifactorial, with several underlying causes, such as chronic irritation resulting from endotracheal intubation, vocal cord injury or trauma, gastroesophageal reflux disease, laryngopharyngeal reflux, chronic cough, laryngeal trauma or can be associated with infectious agents, autoimmune conditions, or iatrogenic factors.^11,12^ Lethal upper airway obstruction is uncommon and often results from an ingested foreign body. Mechanisms of death include not only choking but also arrhythmias or apnea from vagal stimulation, hemorrhage from injury to blood vessels such as the aorta or the heart, and sepsis from more long-standing impaction.^13^ Vocal granuloma, as in the reported case, may also be associated with death from significant occlusion or narrowing of the airways. Airway narrowing may also be intrinsic due to mucosal swelling in anaphylaxis or local or systemic infections such as Ludwig angina, retropharyngeal abscess, acute epiglottitis, or infectious mononucleosis.^14^ External pressure from tumors, hemorrhage, or an unusual neck and head position may also cause critical airway narrowing.^13,15^ Endotracheal tube can cause trauma to the larynx due to a highly large size, prolonged intubation, abnormal position of the patient's head and larynx, improper location or overpressure of the tube cuff, and extubation trauma. They commonly occur in the vocal process of the arytenoid cartilage, where the endotracheal tube exerts pressure. The vocal process is also vulnerable due to its thin mucoperichondrial covering.^16,17^ In our case, the most probable etiology can be attributed to previous multiple intubations and laryngeal injury post endotracheal intubation during the surgical procedure for vocal polyp one month back and loss of follow-up thereafter, leading to the vocal granuloma formation, which further leads to significant airway obstruction.

The prevalence of laryngeal granulomas is relatively low, but their incidence has increased in recent years due to advances in surgical procedures and the increasing use of endotracheal intubation. According to the Farwell grading system, large and extra-large lesions were relatively rare (Grade 3 and Grade 4).^3,4^

Lee et al.^18^ observed that among 13 out of 21 patients, bilateral vocal granulomas were seen in 8 patients, while in the remaining 5 patients, left-sided unilateral vocal granulomas were noted. This phenomenon could be attributed to the right-side holding of the endotracheal tube, causing damage in the left vocal cord. The vocal cord granuloma, in our case, was also found on the left side and can be attributed to the above-mentioned factor, which result from the surgical intervention a month ago. However, detailed documentation was not available.

Vocal process granulomas appear as polypoid or nodular masses in the laryngeal region. They are not granulomatous lesions on light microscopic examination, as they lack prominent aggregates of mononuclear and multinucleated histiocytes. Microscopically, they are hyperplasic fibrous tissue, the same as a benign proliferation of normal tissues, such as skin keloid. They are marked either by an intact epithelial surface or an ulcerated surface, and the adjacent tissues are expanded by granulation tissue along with intense proliferation of blood vessels and inflammatory cell infiltrate represented by polymorphonuclear neutrophils, lymphocytes, occasional histiocytes, and eosinophils. As these lesions age, granulation tissue may be initially replaced by fibrosis and finally by hyperplasia of the superficial squamous epithelium.^7,12,19^

Clinical features of laryngeal granulomas typically include hoarseness, dysphonia, and dyspnea. Patients may also experience coughing, globus sensation, and a persistent need to clear the throat. In some cases, the symptoms may be subtle and overlooked, leading to delayed diagnosis and potential complications. The recurrence rate as high as 90% after the surgical removal of granuloma has been reported.^20^

Laryngeal granulomas are investigated by clinical evaluation, laryngoscopy, and imaging studies such as computed tomography or magnetic resonance imaging to assess the extent of the lesion and to rule out other entities. However, a definitive diagnosis can be given only after a histopathological examination.

Treatment options for laryngeal granulomas include conservative measures such as voice therapy, vocal rest, and pharmacotherapy with proton pump inhibitors. Surgical intervention may be necessary in cases of severe airway compromise or failed conservative management. The surgical approaches range from endoscopic procedures to open surgical resection, depending on the extent and location of the lesion. There is no consensus in the literature regarding the best treatment for laryngeal granulomas due to the diverse etiology. However, microsurgery was the best treatment for post-intubation granulomas, which were the most frequent. All are sensitive to radiotherapy.^3,21,22^

## CONCLUSION

Although rare, laryngeal granulomas can cause fatal asphyxia when the granuloma obstructs the laryngeal airway completely or near-completely, preventing the passage of air into the lungs. Asphyxiation occurs when respiratory distress intensifies, leading to an inadequate oxygen supply and subsequent death. Autopsy plays a vital role in confirming the cause of death in cases of fatal asphyxia due to exuberant laryngeal granuloma by finding evidence of airway obstruction, such as marked narrowing or complete occlusion of the laryngeal lumen and determining the extent of the inflammation and any associated complications that may have contributed to the fatality. In our case, the autopsy helped in concluding the cause of death as asphyxia due to glottic obstruction by a laryngeal granuloma consequent to the surgical removal of a vocal polyp.
